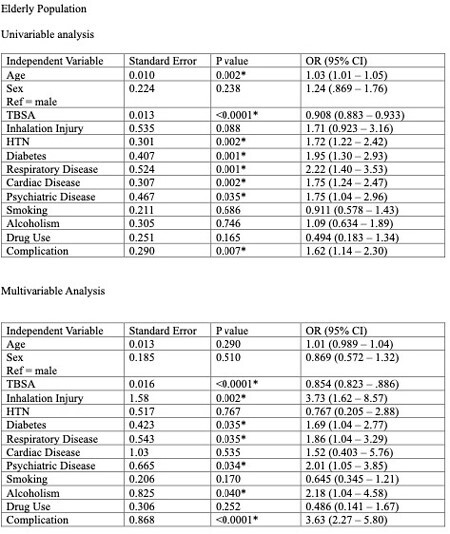# 718 Predictors of Lengthened Admission in Elderly Burn Patients, a Secondary Analysis of 529 Cases

**DOI:** 10.1093/jbcr/irae036.262

**Published:** 2024-04-17

**Authors:** Xi Ming Zhu, Diana Tedesco, Shahriar Shahrokhi, Marc G Jeschke

**Affiliations:** McMaster University, Hamilton, ON; Hamilton Health Sciences Corporation, Hamilton, ON; Hamilton Health Sciences, Hamilton, ON; McMaster University, Hamilton, ON; Hamilton Health Sciences Corporation, Hamilton, ON; Hamilton Health Sciences, Hamilton, ON; McMaster University, Hamilton, ON; Hamilton Health Sciences Corporation, Hamilton, ON; Hamilton Health Sciences, Hamilton, ON; McMaster University, Hamilton, ON; Hamilton Health Sciences Corporation, Hamilton, ON; Hamilton Health Sciences, Hamilton, ON

## Abstract

**Introduction:**

Existing research has examined the relationship between the amount of total body surface area (TBSA) burn and length of stay (LOS) for older adults. As a result, the conventional ratio of 1 day LOS/1% TBSA ratio has been updated to 2 days LOS/1% TBSA. In this study, we aim to elucidate patient and injury characteristics that affect our prognostic indicator, leading to a prolonged LOS.

**Methods:**

This was a secondary analysis of a cohort study of surviving patients admitted to a tertiary adult burn center between January 1, 2006, and June 30, 2021. Older adult patients aged 60 and over were stratified into expected LOS (< 2.0 days/%TBSA) and greater than expected LOS (>2.0 days/%TBSA). Patient demographics, TBSA, inhalation injury, pre-admission co-morbidities, and in-hospital complications were tabulated. Logistic regressions were performed using IBM SPSS Statistics 29 and Stata Statistical Software: Release 18.

**Results:**

529 patients with a mean age of 71 years were included for analysis of the older adult population. 266 patients had the expected LOS/TBSA ratio, and 263 exceeded this ratio. Univariable analysis indicated patients with greater age [1.03 (1.01 – 1.05) p = 0.002], hypertension [1.72 (1.22 – 2.42) p = 0.002], diabetes [1.95 (1.30 – 2.93) p = 0.001], respiratory disease [2.22 (1.40 – 3.53) p = 0.001], cardiac disease [1.75 (1.24 – 2.47) p = 0.002], psychiatric illness [1.75 (1.04 – 2.96) p = 0.035], and those who experienced complications (such as infection, graft failure, pneumonia, sepsis) [1.62 (1.14 – 2.30) p = 0.007] during admission were more likely to exceed their predicted LOS.

Multivariate analysis found that inhalation injury [3.73 (1.62 – 8.57) p = 0.002], diabetes [1.69 (1.04 – 2.77) p = 0.035], respiratory disease [1.86 (1.04 – 3.29) p = 0.035], psychiatric illness [1.50 (1.01 – 1.87) p = 0.04], alcohol use [2.18 (1.04 – 4.58) p = 0.04], and complications [3.63 (2.27 – 5.80) p < 0.0001] were contributing factors that increased the likelihood of patients exceeding predicted LOS. Diabetes was not a statistically significant contributor.

**Conclusions:**

Progress has been made to identify patient and injury factors that increase the likelihood of increased LOS for older adult patients with burn injuries. This provides valuable data for physicians to better assess their patients and improve their quality of care.

**Applicability of Research to Practice:**

Identification of key burn patient characteristics aids in prognostication. Determining length of stay aids with coordination of patient care with the multi-disciplinary team.